# High burden of extrapulmonary tuberculosis and its clinical determinants: a 6-year retrospective analysis of patients at Hargeisa TB Hospital, Somaliland

**DOI:** 10.3389/fpubh.2026.1982502

**Published:** 2026-10-20

**Authors:** Abdirashid Muhumed Guled, Abdulkadir Mohamed Nuh, Mohamed Abdilahi Mohamed, Abdisalam Hassan Muse, Hamse Arab Ali, Hamse Adam Abdi, Mustafe Abdilahi Abdiqadir, Amani Yusuf Yonis, Omran Salih

**Affiliations:** 1 School of Postgraduate Studies and Research, Amoud University, Amoud, Somaliland, Somalia; 2 School of Graduate Studies and Research, Gollis University, Hargeisa, Somaliland, Somalia; 3 Faculty of Medicine and Health Sciences, University of Hargeisa, Hargeisa, Somaliland, Somalia; 4 Faculty of Statistics and Data Science, College of Business, Economics and Statistics, University of Hargeisa, Hargeisa, Somaliland, Somalia

**Keywords:** clinical determinants, extrapulmonary tuberculosis, Hargeisa, pulmonary tuberculosis, retrospective study, Somaliland

## Abstract

**Background:**

Extrapulmonary tuberculosis (EPTB) remains a major diagnostic challenge, particularly in resource-limited settings where tuberculosis services are often centred on pulmonary disease. This study assessed the burden, anatomical distribution, and clinical determinants of EPTB among patients treated at Hargeisa TB Hospital, Somaliland.

**Methods:**

A retrospective facility-based cross-sectional study was conducted using tuberculosis registry data from 2019–2024. All 7,211 registered TB patients were included. Missing data were handled using complete-case analysis, whereby participants with missing values for variables were excluded. Descriptive statistics summarized patient characteristics and EPTB sites, while logistic regression identified factors independently associated with EPTB. Adjusted odds ratios (aORs) with 95% confidence intervals (CIs) were reported.

**Results:**

Of 7,211 patients, 3,123 (43.3%) had EPTB and 4,088 (56.7%) had pulmonary TB. Lymphatic TB was the most common specific anatomical form (25.5%), followed by pleural (17.9%), abdominal (15.8%), bone/spinal (14.1%), and meningeal TB (1.3%). Female sex was independently associated with EPTB (aOR = 1.77; 95% CI: 1.60–1.95). Compared with patients aged 0–14 years, higher odds were observed among those aged 15–34 years (aOR = 1.40; 95% CI: 1.23–1.59), 35–54 years (aOR = 2.11; 95% CI: 1.82–2.44), and ≥55 years (aOR = 1.84; 95% CI: 1.57–2.15). New TB cases were also associated with EPTB (aOR = 1.48; 95% CI: 1.21–1.82), whereas HIV-positive and on ART status was inversely associated (aOR = 0.37; 95% CI: 0.27–0.52). Reporting year was not significant.

**Conclusion:**

EPTB constituted a substantial proportion of the TB burden at Hargeisa TB Hospital. Female sex, older ages, and HIV status were independent predictors. Strengthening EPTB-specific diagnostic pathways, referral systems, imaging, molecular testing, and histopathology is essential for improving case detection and TB control in Somaliland.

## Background of the study

Extrapulmonary tuberculosis (EPTB) remains difficult to diagnose because disease may involve almost any organ, is frequently paucibacillary, and often requires site-specific specimen collection, molecular testing, imaging, cytology, or histopathology rather than routine sputum-based pathways (1). Although EPTB accounts for an important share of the global TB burden, its reported frequency varies widely across settings and is often higher in referral hospitals, where diagnostically complex cases concentrate (2).

This diagnostic challenge is especially important in resource-constrained settings in the Horn of Africa, where access to site-specific investigations and timely referral may be limited (3). Hargeisa TB Hospital is a major tertiary referral facility serving patients from urban and surrounding rural areas of Somaliland. However, localized evidence describing the anatomical spectrum of EPTB and the demographic and clinical factors associated with extrapulmonary rather than pulmonary presentation remains limited. This study therefore assessed the burden, anatomical distribution, temporal pattern, and factors associated with EPTB among patients treated at Hargeisa TB Hospital (4).

EPTB accounts for approximately 15–25% of all TB cases globally, although its frequency varies substantially across settings (5). In high-burden countries, particularly in hospital-based populations, EPTB proportions is substantially higher, reaching 49.8% of TB notifications in a tertiary-care setting in Ethiopia (6). Similarly, in South-Central China, lymphatic and multiple EPTB manifestations remain prevalent among hospitalized cohorts, indicating a substantial burden of paucibacillary disease (7). Historically, EPTB was frequently marginalized in national control programs because surveillance systems prioritized the highly transmissible pulmonary forms (8). However, from the past to the present, a shift has occurred due to the rising prevalence of immunosuppressive conditions and the evolution of diagnostic technology (9, 10). While traditional diagnosis relied on clinical evidence and smear microscopy which often failed in EPTB due to low bacillary loads the introduction of Low-Complexity automated Nucleic Acid Amplification Tests (LCaNAATs) like GeneXpert has revolutionized detection in non-respiratory specimens (11, 12) Contemporary studies now demonstrate that EPTB is no longer a declining entity but an old problem resurging in both low-prevalence areas like Spain and high-endemic regions in Asia and Africa (5, 13). This historical trajectory indicates that EPTB management must move beyond extrapolation from pulmonary protocols to site-specific interventions (14).

The contextual background of this study is focused on Hargeisa TB Hospital, the primary referral facility for tuberculosis in Somaliland. The Horn of Africa faces unique epidemiological pressures, including mobile populations and a healthcare infrastructure recovering from prolonged instability (15). In Somaliland, paucibacillary EPTB cases often present to tertiary facilities at advanced stages due to nonspecific symptoms and a lack of early diagnostic capacity at the primary care level (16). As Hargeisa serves as a central hub for both urban and rural patients, the hospital provides a critical vantage point for evaluating the full clinical spectrum of the disease in a region where paucibacillary patterns may differ significantly from international averages (17). Understanding local distribution is essential for the Somaliland National Tuberculosis Programme to optimize resource allocation and clinical algorithms (18).

The categorization of EPTB is defined by its diverse anatomical sites, with lymph node, spinal, and abdominal involvements being the most frequent phenotypes (6, 19). Key covariates associated with these manifestations include human immunodeficiency virus (HIV) co-infection, which significantly increases the risk of disseminated and meningeal TB (20, 21). Demographic factors also serve as critical predictors; for instance, younger age and male sex have been strongly linked to TB lymphadenitis, whereas spinal TB frequently occurs in middle-aged, economically active populations (19, 22). Furthermore, lifestyle comorbidities such as tobacco and alcohol use are increasingly recognized as significant risk factors for unsuccessful treatment outcomes (23, 24). The emergence of drug-resistant EPTB, particularly multidrug-resistant (MDR) musculoskeletal forms, adds another layer of complexity to the clinical management of these patients (7, 25).

Despite the wealth of data from other regions, a critical gap exists regarding the specific factors predisposing patients to EPTB in Somaliland. Most regional strategies rely on data extrapolated from neighboring Ethiopia or general Sub-Saharan African trends, failing to account for the unique socio-demographic and healthcare-seeking behaviors in Hargeisa (26, 27). The novelty of this study lies in its systematic analysis of clinical and demographic covariates within a specialized Somaliland hospital, providing the first localized evidence of the factors driving extrapulmonary dissemination (28, 29). By addressing the diagnostic challenges and spectrum of disease in this specific geopolitical context, this research fills a void in the global mapping of tuberculosis (30).

The purpose of this study is to describe the clinical spectrum, epidemiology, and associated risk factors of EPTB among patients at Hargeisa TB Hospital. By identifying the most prevalent forms of the disease and the specific demographic profiles of those affected, this study contributes to a more integrated approach to TB care beyond pulmonary disease (31, 32). Ultimately, the findings are intended to inform clinical decision-making, support early recognition algorithms, and contribute to the broader goal of reducing TB-related morbidity and disability in Somaliland in accordance with the End TB Strategy (33). Although previous research from Hargeisa TB Hospital has described tuberculosis treatment outcomes and their determinants, the present study addresses a distinct clinical and epidemiological question. Specifically, it characterizes the phenotype and anatomical distribution of extrapulmonary tuberculosis and identifies demographic and clinical factors associated with extrapulmonary rather than pulmonary presentation across a six-year period. By extending the observation period through 2024 and focusing specifically on EPTB site patterns and associated factors, this study provides a more detailed characterization of extrapulmonary disease in Somaliland than was available from earlier treatment-outcome analyses.

## Methodology

### Study area

The study was conducted at Hargeisa TB Hospital, a specialized tertiary-level facility located in Hargeisa, the capital of Somaliland. As a primary referral center for tuberculosis management, the hospital serves a diverse and high-volume patient population from both urban and surrounding rural areas. The facility is equipped to provide diagnostic services, inpatient care, and outpatient monitoring for various forms of tuberculosis, making it a critical site for monitoring regional epidemiological trends. The geographical and clinical scope of this hospital provides a representative cross-section of the tuberculosis burden in the region, particularly for complex and extrapulmonary cases.

### Study design

This study used a retrospective facility-based cross-sectional design to analyze six years of TB registry data (2019–2024). The design was appropriate for estimating the proportion and clinical distribution of EPTB, examining temporal patterns, and identifying demographic and clinical factors associated with EPTB compared with PTB using routinely collected hospital data.

### Data source

The primary data source for this study consisted of the official tuberculosis registration logs and electronic medical records maintained by Hargeisa TB Hospital. These records are part of the national tuberculosis surveillance system and contain standardized information on patient demographics, clinical history, and laboratory results. Data extraction was performed systematically to ensure accuracy, focusing on all patients diagnosed with any form of tuberculosis during the designated timeframe. The use of secondary data from these established registries ensured a comprehensive capture of all reported cases within the facility.

### Population and sample

The study population included all patients who were diagnosed with and received treatment for tuberculosis at Hargeisa TB Hospital between January 2019 and December 2024. A total of 7,211 tuberculosis cases were identified and included in the final analysis through a total population sampling method. Of these, 4,088 were classified as pulmonary tuberculosis (PTB) and 3,123 were classified as extrapulmonary tuberculosis (EPTB). No sampling techniques were applied, as the study aimed to analyze the complete census of patients registered during the study period to ensure the highest possible statistical power and minimize selection bias.

### Study variables

The primary dependent variable in this study was the type of tuberculosis, categorized as either pulmonary (PTB) or extrapulmonary (EPTB). Independent demographic variables included sex and age, which was categorized into four distinct groups: 0–14 years, 15–34 years, 35–54 years, and 55 years and older. Clinical variables included the case type.

Case type was classified using the Hargeisa TB Hospital registry. A ‘New case’ was a patient recorded as newly diagnosed and registered for the current treatment episode. ‘Previous/Transfer’ included patients with a documented history of previous TB treatment and patients transferred from another TB treatment facility for continuation of care. For transferred patients, classification as PTB or EPTB was based on the diagnosis recorded at Hargeisa TB Hospital upon arrival, following review of the referral information and available clinical and diagnostic findings, rather than solely on the initial diagnosis from the referring facility.

### Diagnosis and classification of EPTB

EPTB was defined as TB involving an anatomical site outside the lung parenchyma or tracheobronchial tree. Diagnosis was based on the best available evidence documented in the hospital records, including microbiological testing (GeneXpert MTB/RIF where an appropriate specimen was available), histopathology, relevant imaging, clinical findings, and physician assessment. GeneXpert testing of appropriate non-respiratory specimens was considered bacteriological confirmation, consistent with WHO TB diagnostic guidance. Cases without documented microbiological confirmation were classified as clinically diagnosed EPTB when the treating physician established the diagnosis based on compatible clinical and/or radiological findings and available investigations. Because this was a retrospective study of routinely collected hospital records, the availability of site-specific diagnostic tests varied between patients.

### Data analysis

The collected data were cleaned, coded, and analyzed using statistical software. The original dataset contained 8,000 patient records. Following data cleaning, 789 records (9.9%) were excluded because of missing or incomplete information in variables required for the analysis. Complete-case analysis was therefore conducted using 7,211 patients (90.1% of the original dataset). Because sufficient information was unavailable for all excluded records, formal comparison between included and excluded patients could not be performed, and potential selection bias due to missing data cannot be excluded. Descriptive statistics, including frequencies and percentages, were used to summarize the demographic and clinical characteristics of the study population. To compare the distribution of these characteristics between PTB and EPTB patients, the Chi-square test was employed. To identify factors independently associated with EPTB, both univariate and multivariate logistic regression analyses were conducted. Results were presented as Odds Ratios (OR) and adjusted Odds Ratios (aOR) with their corresponding 95% confidence intervals (CI). A *p*-value of less than 0.05 was considered statistically significant for all tests. Trends over the six-year period were visualized using bar charts and pie charts to illustrate annual fluctuations and anatomical distributions. Multicollinearity was assessed using variance inflation factors (VIF).

### Multicollinearity diagnostics of variables

The mean VIF was 2.07, with individual VIFs ranging from 1.05 to 5.90. All variables had VIF values below the commonly used threshold of 10, indicating no substantial multicollinearity among the predictors (Table 1).

**Table 1:** Multicollinearity diagnostics of variables.

Variable	Category	VIF
Sex	Female	1.57
Age group	15–34	2.40
	35–54	1.79
	55+	1.57
New case	New cases	5.90
HIV status	Positive and on ART	1.05
Source year	2020	1.70
	2021	1.73
	2022	1.75
	2023	1.69
	2024	1.66
Mean VIF		2.07

### Ethical considerations

Ethical approval for the study was obtained from Amoud University institutional review board and the management of Hargeisa TB Hospital. Because the research involved a retrospective analysis of secondary data, the requirement for individual patient consent was waived; however, strict measures were taken to ensure the confidentiality and anonymity of all patient information. No identifiable personal information, such as names or addresses, was extracted or included in the final dataset. Data access was restricted to the research team, and the findings were reported in aggregate form to ensure that individual patients could not be identified, adhering to the ethical principles of the Declaration of Helsinki.

## Result

Figure 1 illustrates the annual trend in extrapulmonary tuberculosis (EPTB) cases at Hargeisa TB Hospital from 2019 to 2024. EPTB cases fluctuated over the study period but showed an overall declining pattern, with the highest number recorded in 2019 and lower-case counts observed toward the end of the study period.

**Figure 1:**
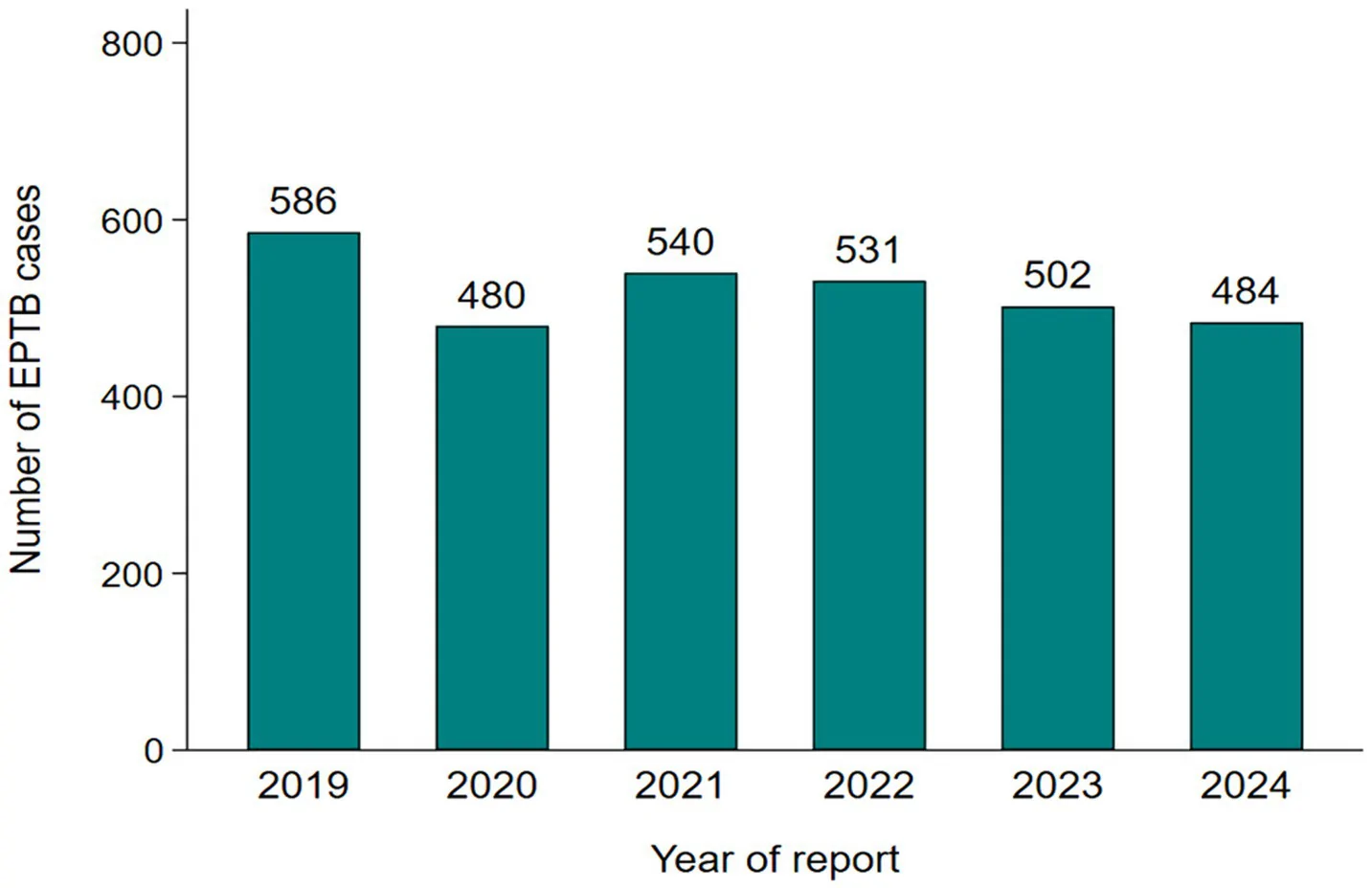
Annual trend in extrapulmonary tuberculosis cases at Hargeisa TB Hospital, 2019–2024.

Figure 2 compares the annual distribution of pulmonary tuberculosis (PTB) and extrapulmonary tuberculosis (EPTB) cases during the study period. PTB consistently accounted for more cases than EPTB across all years. Although both forms showed year-to-year fluctuations, their case numbers generally declined toward the end of the study period, while the relative pattern between PTB and EPTB remained broadly consistent.

**Figure 2:**
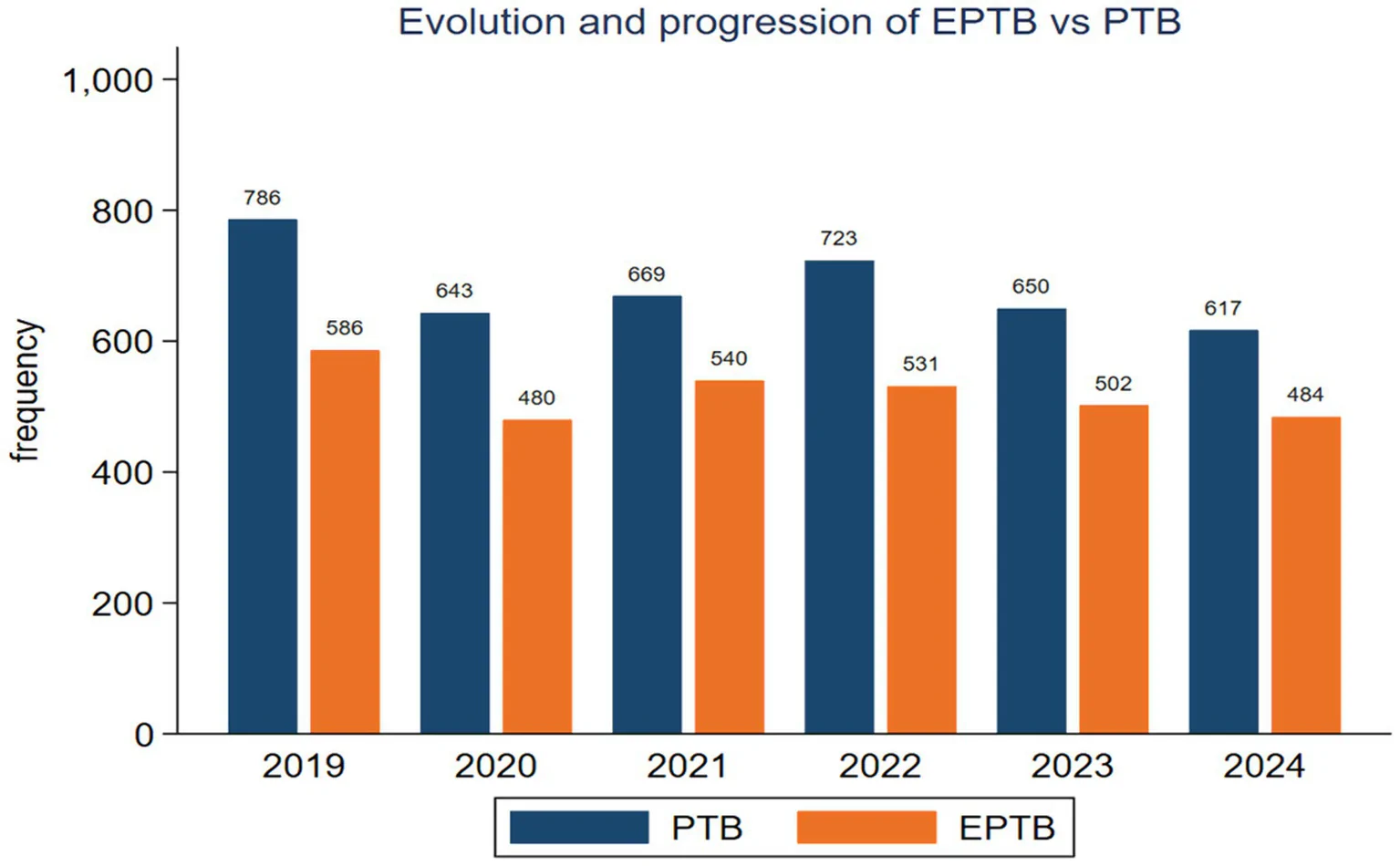
Annual distribution of pulmonary and extrapulmonary tuberculosis cases at Hargeisa TB Hospital, 2019–2024.

### Anatomical distribution of extrapulmonary tuberculosis cases at Hargeisa TB hospital

Figure 3 illustrates the anatomical distribution of EPTB cases. Lymphatic TB was the most common specific site (25.5%), while the aggregated “Other” category accounted for 25.4%. Within the “Other” category, the predominant subtypes were CNS tuberculoma (6.0%), intestinal/peritoneal TB (4.8%), and peripheral bone/joint TB (4.7%), with the remaining proportion comprising several less frequent anatomical sites, as detailed in Figure 3. Overall, the findings demonstrate that EPTB involved a broad range of anatomical sites, with lymphatic and less readily classifiable presentations accounting for approximately half of cases.

**Figure 3:**
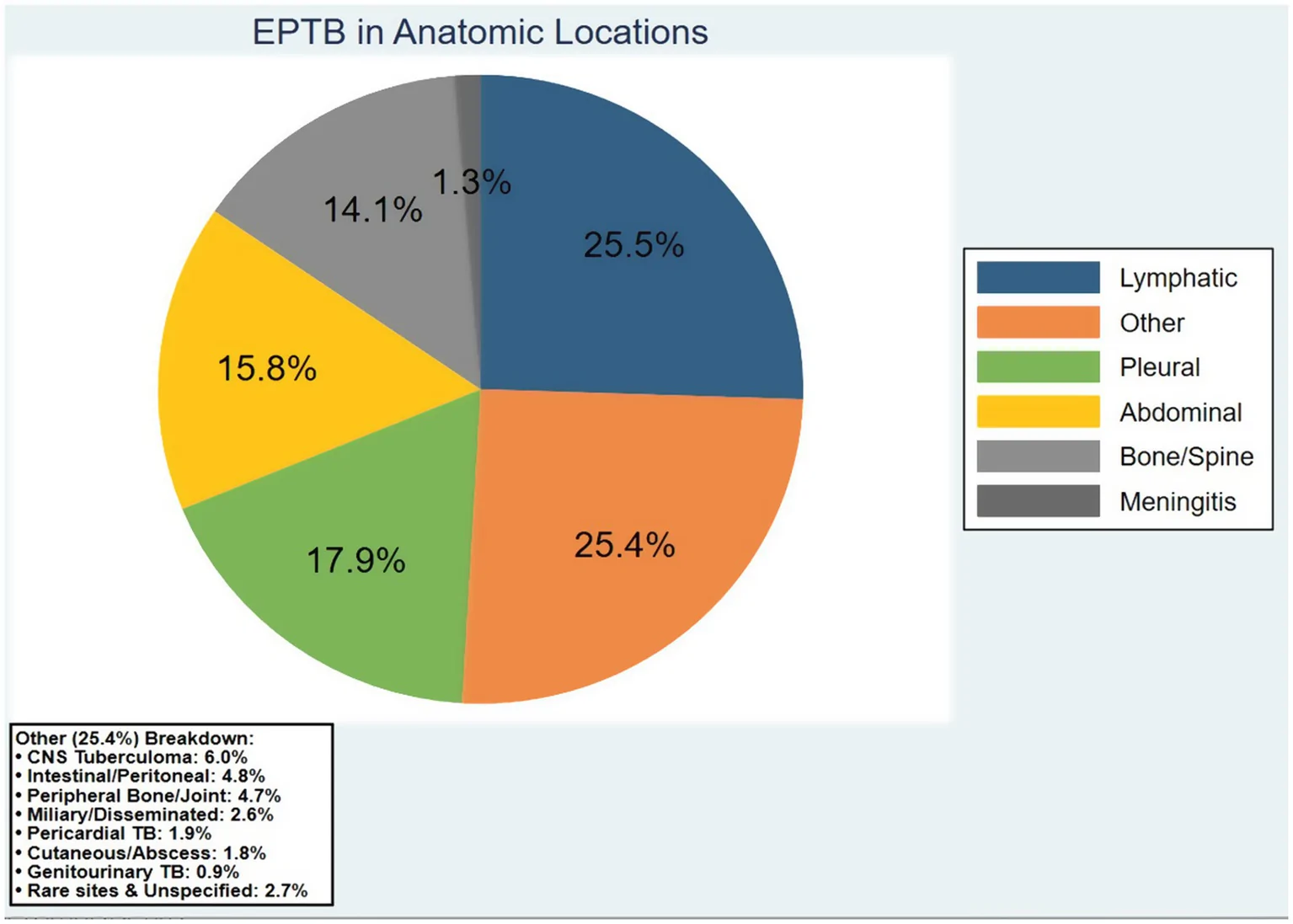
Anatomical distribution of extrapulmonary tuberculosis cases at Hargeisa TB Hospital, 2019–2024.

Among 7,211 TB patients, 4,088 (56.7%) had PTB and 3,123 (43.3%) had EPTB. Significant differences were observed by sex, age, and HIV status. PTB was more common among males (67.5%), whereas females represented a larger proportion of EPTB cases (45.0%). EPTB was less common among children aged 0–14 years (18.4% vs. 25.5% for PTB) but more frequent among patients aged 35–54 years (25.3%) and ≥55 years (17.9%). Most patients were new cases, with a slightly higher proportion among EPTB patients (94.8% vs. 92.9%). HIV positivity was higher among PTB than EPTB patients (3.5% vs. 1.7%). No significant difference was observed in the distribution of PTB and EPTB cases across study years, indicating a relatively stable case distribution over the six-year period (Table 2).

**Table 2:** Comparison of demographic, clinical, and microbiological characteristics between PTB and EPTB patients (2019–2024).

Characteristic	Pulmonary TB (*N* = 4,088)	Extrapulmonary TB (*N* = 3,123)	Total (*N* = 7,211)	*p*-value
Sex				<0.001
Male	2,761 (67.5%)	1,716 (55.0%)	4,477 (62.1%)	
Female	1,327 (32.5%)	1,407 (45.0%)	2,734 (37.9%)	
Age group				<0.001
0–14 years	1,044 (25.5%)	576 (18.4%)	1,620 (22.5%)	
15–34 years	1,677 (41.0%)	1,198 (38.4%)	2,875 (39.9%)	
35–54 years	774 (18.9%)	789 (25.3%)	1,563 (21.7%)	
55 + years	593 (14.5%)	560 (17.9%)	1,153 (16.0%)	
Case type				<0.001
New Case	3,796 (92.9%)	2,959 (94.8%)	6,755 (93.7%)	
Previous/Transfer	292 (7.1%)	164 (5.3%)	456 (6.3%)	
HIV status				<0.001
Negative	3,946 (96.5%)	3,071 (98.3%)	7,017 (97.3%)	
Positive and on ART	142 (3.5%)	52 (1.7%)	194 (2.7%)	
Source year				0.856
2019	786 (19.2%)	586 (18.8%)	1,372 (19.0%)	
2020	643 (15.7%)	480 (15.4%)	1,123 (15.6%)	
2021	669 (16.4%)	540 (17.3%)	1,209 (16.8%)	
2022	723 (17.7%)	531 (17.0%)	1,254 (17.4%)	
2023	650 (15.9%)	502 (16.1%)	1,152 (16.0%)	
2024	617 (15.1%)	484 (15.5%)	1,101 (15.3%)	

The univariate and multivariate analyses identify several key demographic and clinical factors significantly associated with the presentation of Extra-Pulmonary Tuberculosis (EPTB) compared to Pulmonary Tuberculosis (PTB). According to the multivariate analysis, female patients are 77% more likely than males to be diagnosed with EPTB (aOR 1.77, 95% CI 1.60–1.95, p<0.001 ). Age also serves as a significant predictor, with all age groups over 14 years showing increased odds of EPTB compared to the 0–14 pediatric baseline; the strongest association is observed in the 35–54 age group, who are more than twice as likely to have EPTB (aOR 2.11, 95% CI 1.82–2.44, p<0.001 ). Clinical status further influences the diagnosis, as new cases are significantly more likely to present with EPTB than those with a history of previous treatment or transfer (aOR 1.48, 95% CI 1.21–1.82, p<0.001 ). Interestingly, HIV-positive and on ART were significantly less likely to be diagnosed with EPTB compared to HIV-negative individuals in this study population (aOR 0.37, 95% CI 0.27–0.52, p<0.001 ). Consistent with earlier findings, the year of reporting showed no statistically significant impact on the likelihood of presenting with EPTB, indicating that the proportional risk factors remained stable across the six-year (Table 3).

**Table 3:** Univariate and multivariate analysis of clinical and demographic characteristics associated with EPTB.

Characteristics	Univariate analysis			Multivariate analysis		
	OR	95% CI	*p*-value	aOR	95% CI	*p*-value
Sex						
Males	1. Ref			1. Ref		
Female	1.71	[1.55, 1.88]	<0.001	1.77	[1.60, 1.95]	<0.001
Age group						
0–14 years	1. Ref			1. Ref		
15–34 years	1.29	[1.14, 1.47]	<0.001	1.40	[1.23, 1.59]	<0.001
35–54 years	1.85	[1.60, 2.13]	<0.001	2.11	[1.82, 2.44]	<0.001
55 + years	1.71	[1.47, 2.00]	<0.001	1.84	[1.57, 2.15]	<0.001
Case type						
Previous/Transfer	1. Ref			1. Ref		
New case	1.39	[1.14, 1.69]	<0.001	1.48	[1.21, 1.82]	<0.001
HIV status						
Negative	1. Ref			1. Ref		
Positive and on ART	0.47	[0.34, 0.65]	<0.001	0.37	[0.27, 0.52]	<0.001
Source year						
2019	1. Ref			1. Ref		
2020	1.00	[0.85, 1.17]	0.987	0.97	[0.82, 1.14]	0.702
2021	1.08	[0.93, 1.27]	0.318	1.06	[0.90, 1.24]	0.479
2022	0.99	[0.84, 1.15]	0.849	0.97	[0.83, 1.13]	0.694
2023	1.04	[0.88, 1.21]	0.662	0.99	[0.84, 1.16]	0.876
2024	1.05	[0.90, 1.23]	0.533	0.98	[0.83, 1.16]	0.825

## Discussion

In the present six-year retrospective analysis of patients managed at Hargeisa TB Hospital, 43.3% of all registered tuberculosis cases were classified as extrapulmonary tuberculosis (EPTB). This finding demonstrates a remarkably high clinical burden which is more than four in every ten TB patients treated at the hospital had disease outside the pulmonary parenchyma. This is particularly important because EPTB is diagnostically challenging, frequently paucibacillary, can involve almost any organ, and may require imaging, histopathology, fine-needle aspiration, biopsy, or molecular testing of non-respiratory specimens rather than conventional sputum-based diagnostic pathways (34). The observed proportion is strikingly consistent with an earlier five-year retrospective study conducted at the same Hargeisa TB Hospital during 2019–2023, which reported EPTB in 43.2% of 6,069 patients with complete records (4). The proportion of EPTB observed in the present study was comparable with hospital-based findings from Ethiopia, where 40.1% of TB patients in Addis Ababa (35) and 38.8% in Harar (36) had EPTB. Similarly, a five-year retrospective study from Bosaso TB Hospital reported 36.1% (37). The slightly higher proportion in Hargeisa may reflect differences in referral patterns, diagnostic capacity, patient case-mix, healthcare-seeking behaviour, and case ascertainment. In contrast, the present estimate was lower than the 49.8% reported from a teaching hospital in Addis Ababa (6).

The Hargeisa burden was also higher than estimates from broader regional and national studies. A nationwide multicentre study in Pakistan reported 29.2% EPTB (37), while a systematic review and meta-analysis of 68 African studies estimated an overall prevalence of 26%, increasing to 32% in Eastern Africa (2). Lower proportions were also reported among more than 53,000 TB patients in Tanzania (17.6%) (38) and more than 10,000 patients in Kilifi County, Kenya (12%) (39). these variations highlight substantial geographical heterogeneity in EPTB burden, likely influenced by differences in referral systems, diagnostic resources, patient characteristics, HIV epidemiology, and surveillance practices. The high proportion of EPTB observed in this study should be interpreted in the context of referral bias. As a specialized tertiary referral center, Hargeisa TB Hospital is more likely to receive complex, atypical, and diagnostically challenging EPTB cases than primary care facilities. This concentration of referred cases may inflate the observed EPTB proportion relative to primary care or community-level estimates; therefore, the high percentage observed in this study should be regarded as a hospital-based proportion rather than a population-level prevalence estimate for Somaliland. The high burden of EPTB has important implications for TB control in Somaliland, as reliance on cough screening, chest radiography, and sputum testing alone may miss a substantial proportion of cases. TB services should therefore strengthen EPTB-specific diagnostic pathways, including timely referral, appropriate extrapulmonary specimen collection, molecular testing, imaging, cytology, and histopathology. Diagnostic capacity may also have influenced EPTB case ascertainment during the study period. Diagnosis relied on available GeneXpert MTB/RIF testing of appropriate non-respiratory specimens, histopathology, imaging, and clinical assessment (13, 40). However, year-specific availability and utilization of these modalities, including GeneXpert Ultra, were not consistently documented. Because EPTB is frequently paucibacillary and diagnostically challenging, changes in access to molecular testing, histopathology, and imaging could have influenced case detection; therefore, the observed temporal patterns may partly reflect variation in diagnostic ascertainment rather than true changes in disease occurrence (34). Female was independently associated with extrapulmonary tuberculosis in the present study. This finding is consistent with evidence from the United States (41), Spain (42), China (43), European surveillance data (44), and Ethiopia (45), where female TB patients were also more likely to present with extrapulmonary rather than pulmonary disease. The consistency across diverse epidemiological settings suggests that sex may influence the clinical phenotype of tuberculosis. Proposed explanations include sex-related differences in immune responses, hormonal regulation, anatomical patterns of disease, behavioural exposures, healthcare-seeking behaviour, and referral pathways.

Age was also significantly associated with EPTB, with showing a greater likelihood of extrapulmonary presentation than children. Similar associations with older or middle-aged populations have been reported in Spain and other clinical settings (5, 42). However, studies from China (43), Europe (44), and Pakistan (37) have reported higher EPTB occurrence among younger patients and children. These contrasting findings demonstrate that the relationship between age and EPTB is not uniform across populations. Differences in the anatomical distribution of EPTB, immune status, comorbidities, population structure, diagnostic capacity, and referral patterns may explain this heterogeneity. The prominent association among adults in Hargeisa may therefore reflect a locally distinct clinical and referral profile rather than a universal biological age effect. HIV-positive and on ART showed an inverse association with EPTB in the present study. This finding contrasts with much of the established literature, in which HIV infection, particularly advanced immunosuppression, has been associated with extrapulmonary and disseminated tuberculosis (21, 46). The apparent discrepancy may be explained by the combined classification of HIV positivity and on ART exposure. HIV-associated immunosuppression increases the potential for dissemination of *Mycobacterium tuberculosis*, whereas effective antiretroviral therapy restores cell-mediated immunity and may reduce the occurrence of extrapulmonary disease. Evidence from Ethiopia has demonstrated increased EPTB risk among individuals with advanced HIV disease and severe immunosuppression, while ART was associated with a substantially reduced risk (27). However, this inverse association between HIV-positive and on ART status and EPTB should be interpreted cautiously. Because HIV-positive and on ART exposure were recorded as a combined variable, their independent effects could not be disentangled. This finding should therefore not be interpreted as evidence of a protective effect of HIV infection and may reflect differences in treatment exposure, timing of diagnosis, clinical management, or residual confounding. Separate analyses of HIV status and ART exposure were not possible because these variables were not consistently recorded independently in the retrospective dataset. Future prospective studies should separately measure HIV status, ART initiation and duration, and CD4 counts to better distinguish the effects of HIV-related immunosuppression from those of antiretroviral therapy on EPTB presentation. This distinction would allow a more accurate assessment of the relationship between HIV disease severity, treatment status, and extrapulmonary involvement.

## Conclusion

Extrapulmonary tuberculosis represents a major and persistent component of the TB burden at Hargeisa TB Hospital, with female sex and older ages emerging as important independent determinants. These findings underscore the need to move beyond a predominantly pulmonary-focused diagnostic approach in Somaliland. Strengthening early recognition of EPTB, referral pathways, molecular testing, imaging, cytology, histopathology, and site-specific specimen diagnosis should be prioritized within routine TB services. At the policy level, strengthening EPTB screening and clinical suspicion at primary care facilities, supported by clear referral criteria and timely access to higher-level diagnostic services, is essential to promote earlier detection and reduce delayed referrals. Future multicentre prospective studies are recommended to validate these determinants, clarify the role of HIV and ART separately, and define site-specific patterns of EPTB to support more precise, equitable, and effective tuberculosis control.

### Limitations

1. An important limitation is the absence of several potentially relevant clinical confounders, including diabetes, malnutrition, smoking, previous immunosuppressive therapy, and other comorbidities, which were not consistently captured in the retrospective records and could not therefore be included in the regression model.
2. HIV and ART could not be independently evaluated. Because HIV status and ART exposure were recorded as a combined variable, their independent associations with EPTB could not be disentangled. This is particularly important when interpreting the observed HIV-related association.
3. Although patients were referred from different geographic areas, this remains a hospital-based study and therefore cannot be interpreted as a nationally representative estimate of EPTB prevalence across Somaliland.
4. The exclusion of 789 records (9.9%) because of missing or incomplete data may have introduced selection bias, particularly because a formal comparison between included and excluded records could not be performed

## Data Availability

The original contributions presented in the study are included in the article/supplementary material, further inquiries can be directed to the corresponding author.
